# Free radical degradation in aqueous solution by blowing hydrogen and carbon dioxide nanobubbles

**DOI:** 10.1038/s41598-021-82717-z

**Published:** 2021-02-04

**Authors:** Toyohisa Fujita, Hiromi Kurokawa, Zhenyao Han, Yali Zhou, Hirofumi Matsui, Josiane Ponou, Gjergj Dodbiba, Chunlin He, Yuezou Wei

**Affiliations:** 1 College of Resources, Environment and Materials, Guangxi University, Nanning, 530004 China; 2 Faculty of Medicine, The University of Tsukuba, Tsukuba, 305-8575 Japan; 3 Graduate School of Engineering, The University of Tokyo, Tokyo, 113-8656 Japan

**Keywords:** Chemical biology, Environmental sciences, Medical research, Chemistry, Engineering, Nanoscience and technology

## Abstract

The main findings are the hydroxyl radical scavenging and the superoxide anion diminishing by mixing the carbon dioxide (CO_2_) nanobubbles after hydrogen nanobubble blowing in water and alcohol aqueous solution. The nanobubbles produce the hydroxyl radical by ultrasonic waves, changing the pH and catalyst and so on, while the nanobubble is very reactive to scavenge free radicals. In this research especially hydrogen (4% H_2_ in argon) and CO_2_ nanobubbles have been blown into hydrogen peroxide (H_2_O_2_) added pure water, ethanol, and ethylene glycol aqueous solution through a porous ceramic sparger from the gas cylinder. The aqueous solutions with H_2_O_2_ are irradiated by ultraviolet (UV) light and the produced hydroxyl radical amount is measured with spin trapping reagent and electron spin resonance (ESR). The CO_2_ nanobubble blowing extremely has reduced the hydroxyl radical in water, ethanol, and ethylene glycol aqueous solution. On the other hand, when H_2_ nanobubbles are brown after CO_2_ nanobubble blowing, the hydroxyl radical amount has increased. For the disinfection test, the increase of hydroxyl radicals is useful to reduce the bacteria by the observation in the agar medium. Next, when the superoxide anion solution is mixed with nanobubble containing water, ethanol, and ethylene glycol aqueous solution, H_2_ nanobubble has reduced the superoxide anion slightly. The water containing both CO_2_ and H_2_ nanobubble reduces the superoxide anion. The less than 20% ethanol and the 30% ethylene glycol aqueous solution containing CO_2_ nanobubbles generated after H_2_ nanobubble blowing can diminish the superoxide anion much more. While the H_2_ nanobubble blowing after CO_2_ nanobubble blowing scavenges the superoxide anion slightly. The experimental results have been considered using a chemical reaction formula.

## Introduction

Reactive oxygen species are hydroxyl radical (∙OH), superoxide anion (∙O_2_^−^), hydrogen peroxide (H_2_O_2_), single oxygen (^1^O_2_), and ozone (O_3_), while hydroxyl radical (∙OH) and superoxide anion (∙O_2_^−^) are free radical. This report is not the free radical production by nanobubble but describes the free radical degradation in aqueous solution by adding nanobubbles. Various kinds of materials have been reported to scavenge free radicals in medical and pharmacy fields in the paper such as FREE RADICAL RESEARCH and so on. However, there are a few reports for free radical degradation by adding and mixing nanobubbles, while, there are several reports for the detection and production of free radicals by nanobubbles. The aim of this research is how to reduce or increase the existing free radical by nanobubble, therefore, in the beginning, the reports of free radical production conditions by nanobubble are investigated. Takahashi et al. reported that the hydroxyl radical generation with ESR measurement using 5,5-dimethyl-1-pyrroline N-oxide (DMPO) as a spin-trapping reagent has been observed after collapsing of high concentration air microbubbles in water produced by a pump through a gas-dissolution tank and a microbubble-generating nozzle^1^. Next, his group reported that the ∙OH producing in both cases of air (oxygen microbubbles and nitrogen microbubbles) in the acidic condition at pH 2 and 3^2^ and the generation of hydroxyl radicals from the collapse of oxygen or air microbubbles was markedly enhanced by the existence of copper ion catalyst by the copper wire under pH2.2 acidic HCl solution^3^. Also, the microbubbles on ozonized water indicated the presence of hydroxyl radical by the collapse of microbubble^4^. Recent papers are referred the above papers^5^ and the oxygen nanobubble stability was confirmed to be pH-dependent and the collapsed oxygen nanobubble generate the free radical that induced the photodegradation of oxytetracycline^6^. When the absolute value of zeta potential of nanobubble is low, there is a possibility to collapse of nanobubble by Brownian motion and produce the free radical and also the existence of catalysts enhance the radical generation. Ozone addition can produce the hydroxyl radical. On the other hand, the free radical could not generate by the self-collapse of air micro-nano bubbles in pure water produced by fiber membrane filter, and the hydroxyl radical peak was observed with weak supersonic wave^7^. The radical production by ultrasonic wave irradiation becomes more important to produce the radicals, especially hydroxyl radicals in water by comparing no irradiation and irradiation^8^ and the ultrasonic waves collapse the nanobubble (hydrogen) and increase the temperature of water^9^. Therefore, the pure water and alcohol mixed water that does not change the pH and not irradiated an ultrasonic wave is utilized in this report. As the application of produced radicals, there are reports for environmental cleaning related to wastewater treatment^10–13^ and the medical application by reducing oxygen molecules in a chaotic manner within the tumour^14^. In this paper, the bacteria reduction is also examined when the hydroxyl radical enhances in the aqueous solution.

On the other hand, the nanobubble effects to degrade the existing free radicals have been investigated for recent 10 years. In 2010 the nano-bubble hydrogen-dissolved water, which was prepared using a microporous-filter hydrogen-jetting device, scavenged reactive oxygen species (ROS) indispensable to slightly exist as a signal for tumor cell growth^15^. In 2014 the same group reported that Oxygen nanobubble improved blood oxygenation, however, microbubbles also cause tissue damage as well as free radical production and that oxygen itself can be toxic^16^. In 2015 the hydrogen nanobubbles produced by gas–liquid two-phase flow swivel apparatus and the antioxidant activity of nano-bubble hydrogen dissolved water were investigated by the DMPO-spin trap ESR in the H_2_O_2_–UVB irradiation system or 2,2′-bipyridyl method. The hydrogen nanobubbles could reduce the hydroxyl radical concentration^17^. It has been reported in 2018 that the hydrogen nanobubble water can effectively remove cytotoxic reactive oxygen species (ROS) such as ∙OH, ClO–, ONOO–, and∙ O_**2**_^−^ both in vivo and in vitro^18^. Cancer cell growth was inhibited in the hydrogen nanobubble-containing medium compared to the non-containing medium (in vitro)^19^. They have reported mainly the scavenging of ROS by using hydrogen nanobubbles.

In this experiment, the degradation of free radicals for hydroxyl radical and superoxide anion have been compared with single nanobubbles (H_2_ + Ar, CO_2_, N_2_, O_2_) and mixture nanobubbles (H_2_ + Ar and CO_2_) in water and alcohol aqueous solution. The hydroxyl radical in free radical included aqueous solution (pure water, ethanol, and ethylene glycol) is prepared by H_2_O_2_ addition followed by blowing various nanobubble into the liquid, and the hydroxyl radical scavenging is investigated for single nanobubbles and mixture nanobubbles. The superoxide anion in free radical-induced aqueous solution is prepared by a hypoxanthine (HX) and xanthine oxidase (XO) system and mixed with nanobubble included aqueous solution. The superoxide anion scavenging is examined using the single nanobubbles and mixture nanobubbles. The free radical concentration is measured with ESR by using G-CYPMPO as a spin trapping reagent. The degradation of free radicals is effective for healthy beverage and the increase of hydroxyl radical eliminates the bacteria concentration.

## Results

### Produced nanobubbles size in aqueous solution

The mean diameter of produced nanobubble in various aqueous solutions are listed in Table 1. The mean diameter of H_2_ (4% in Ar), CO_2_, O_2,_ and N_2_ nanobubble in water is between 100 and 200 nm. The mean diameter of H_2_ (4% in Ar) in ethanol aqueous solution is between 150 and 250 nm, while in the ethylene glycol aqueous solution the nanobubble diameter becomes larger between 500 and 1000 nm. The mean diameter of CO_2_ nanobubble in ethanol solution is between 250 and 300 nm and in the ethylene glycol aqueous solution the nanobubble diameter becomes larger between 400 and 1000 nm similar to H_2_ (4% in Ar) nanobubble in ethylene glycol. The mean diameter of nanobubble by both blowing H_2_ (4% in Ar) gas and next CO_2_ gas in ethanol aqueous solution is about 200 nm, however, the mean diameter of nanobubble by CO_2_ gas and next H_2_ (4% in Ar) gas becomes larger between 200 and 500 nm. H_2_ (4% in Ar) nanobubble size in water is gradually increased and stable about 400 nm after 10 days and exists more than 100 days. While the CO_2_ nanobubble is gradually increased and disappeared after several days. In Table 1 the solubility of the gas in water is also listed^20^. The CO_2_ gas solubility is one order larger comparing other gas like H_2_, Ar, O_2,_ and N_2_.

**Table 1:** Mean diameters of the nanobubble produced by blowing gases into water, ethanol, and ethylene glycol solution through porous ceramics for every 30 min.

	Mean diameter, nm	Solubility vol/cm^3^ at 1 atm and 20 °C^20^
H_2_ (4% in Ar) gas into water	130	H_2_ 0.018, Ar 0.035
H_2_ (4% in Ar) gas into 10% ethanol and 90% water	230	
H_2_ (4% in Ar) gas into 20% ethanol and 80% water	180	
H_2_ (4% in Ar) gas into 50% ethanol and 50% water	150	
H_2_ (4% in Ar) gas into 30% ethene glycol and 70% water	570	
H_2_ (4% in Ar) gas into 50% ethene glycol and 50% water	820	
H_2_ (4% in Ar) gas and next CO_2_ gas into water	140	
H_2_ (4% in Ar) gas and next CO_2_ gas into 10% ethanol and 90% water	200	
H_2_ (4% in Ar) gas and next CO_2_ gas into 20% ethanol and 80% water	220	
H_2_ (4% in Ar) gas and next CO_2_ gas into 50% ethanol and 50% water	200	
CO_2_ gas into water	115	CO_2_ 0.88
CO_2_ gas into 10% ethanol and 90% water	250	
CO_2_ gas into 20% ethanol and 80% water	280	
CO_2_ gas into 50% ethanol and 50% water	260	
CO_2_ gas into 30% ethene glycol and 70% water	430	
CO_2_ gas into 50% ethene glycol and 50% water	730	
CO_2_ gas into water and then H_2_ (4% in Ar) gas into water	130	
CO_2_ gas and next H_2_ (4% in Ar) gas into 10% ethanol and 90% water	200	
CO_2_ gas and next H_2_ (4% in Ar) gas into 20% ethanol and 80% water	460	
CO_2_ gas and next H_2_ (4% in Ar) gas into 50% ethanol and 50% water	400	
O_2_ gas in water	150	O_2_ 0.031
N_2_ gas in water	180	N_2_ 0.016

### ESR measurement of free radicals mixed with nanobubble aqueous solution

**Figure 1:**
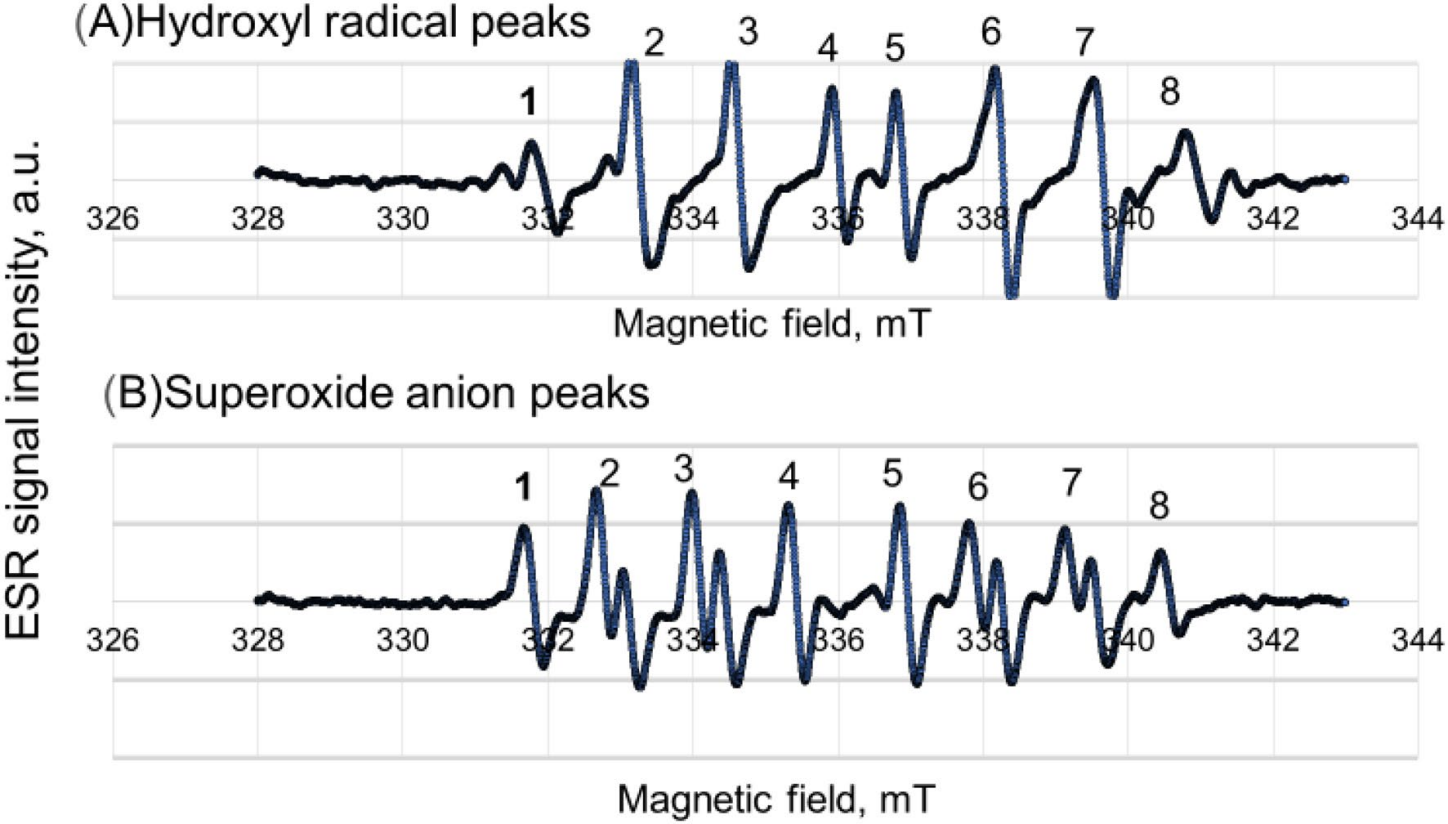
Comparison between hydroxyl radical (**A**) and superoxide anion (**B**) peaks.

The ESR spin adducts of sc-5-(5,5-dimethyl-2-oxo-1,3,2-dioxapho-sphinan-2-yl)-5 methyl-1-pyrroline N-oxide (G-CYPMPO) for hydrogen peroxide aqueous solution (0.1 wt%) under 10 s UV-illumination (A) and hypoxanthine/xanthine oxidase (HX/XO) system (B) is shown in Fig. 1. Eight peaks are appeared by CYPMPO, however, the peak positions of two kinds of radicals in the magnetic field are different. In the following figures of this paper, the heights in the assigned number of eight peaks are compared instead of wave patterns of two radicals (A) and (B).

#### Free radical degradation in water

**Figure 2:**
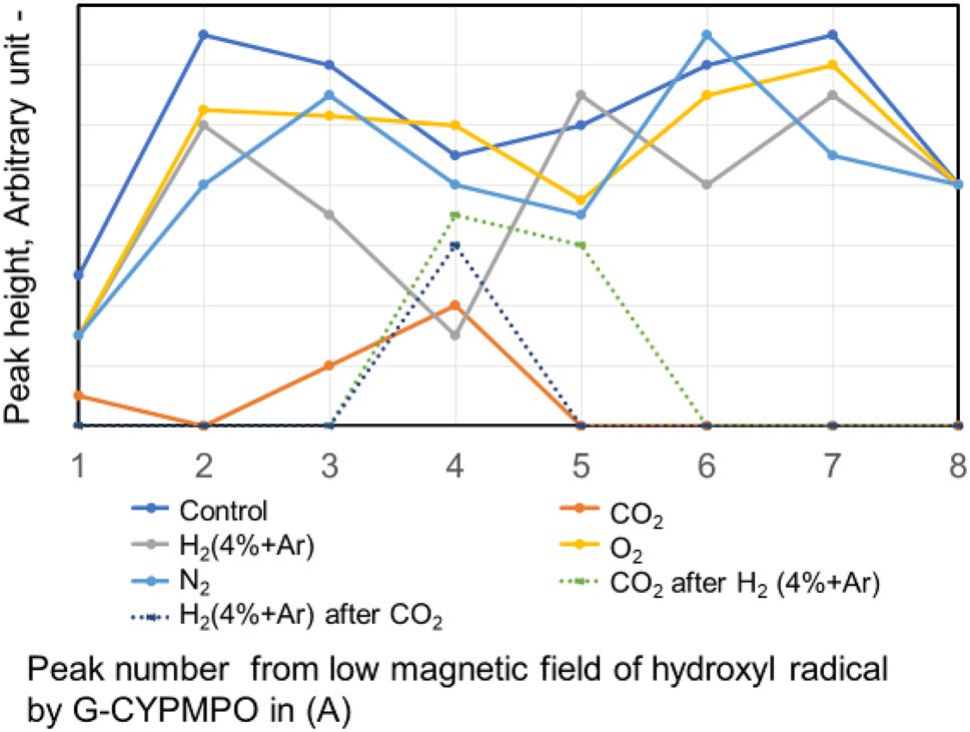
Comparison between different kinds of gas nanobubble peak height of peak number from low magnetic field shown in (A) of Fig. 1 of hydroxyl radical by G-CYPMP.

**Figure 3:**
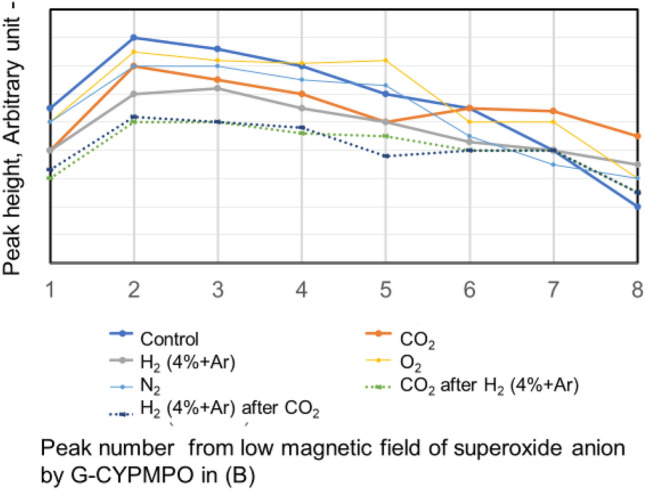
Comparison between different kinds of gas nanobubble peak height of peak number of superoxide anions from low magnetic field shown in (B) of Fig. 1 of superoxide anion radical by G-CYPMPO.

Degradation of hydroxyl radical with ultraviolet in water blown different kinds of nanobubbles N_2_, O_2_, H_2_ (4% in Ar), and CO_2_ are measured by ESR and shown in Fig. 2. For all peaks of hydroxyl radical in water, only CO_2_ nanobubble has decreased compared with the control water and the water containing other nanobubbles. Also, the 4th peak of the water containing H_2_ (4% in Ar) nanobubble has decreased. Therefore, the water containing CO_2_ nanobubble is used for the hydroxyl radical degradation experiment in other aqueous solutions. On the other hand, the degradation by nanobubble combination of CO_2_ after H_2_ (4% in Ar) nanobubble in water and combination of H_2_ (4% in Ar) after CO_2_ nanobubble in water show the similar to the degradation by CO_2_ bubbling. Degradation of superoxide anion radical in the water containing different kinds of nanobubbles N_2_, O_2_, H_2_ (4% in Ar), and CO_2_ are measured by ESR and shown in Fig. 3. The peaks of water blown H_2_ (4% in Ar) nanobubble shows larger degradation peaks comparing with the control water. Therefore, the water bubbled H_2_ (4% in Ar) nanobubble is used for the superoxide anion radical degradation in the following experiment. Both degradation by nanobubble combination of CO_2_ after H_2_ (4% in Ar) nanobubble and combination of H_2_ (4% in Ar) after CO_2_ nanobubble show a little bit larger degradation of H_2_ (4% in Ar).

#### Hydroxyl radical degradation in alcohol aqueous solution

**Figure 4:**
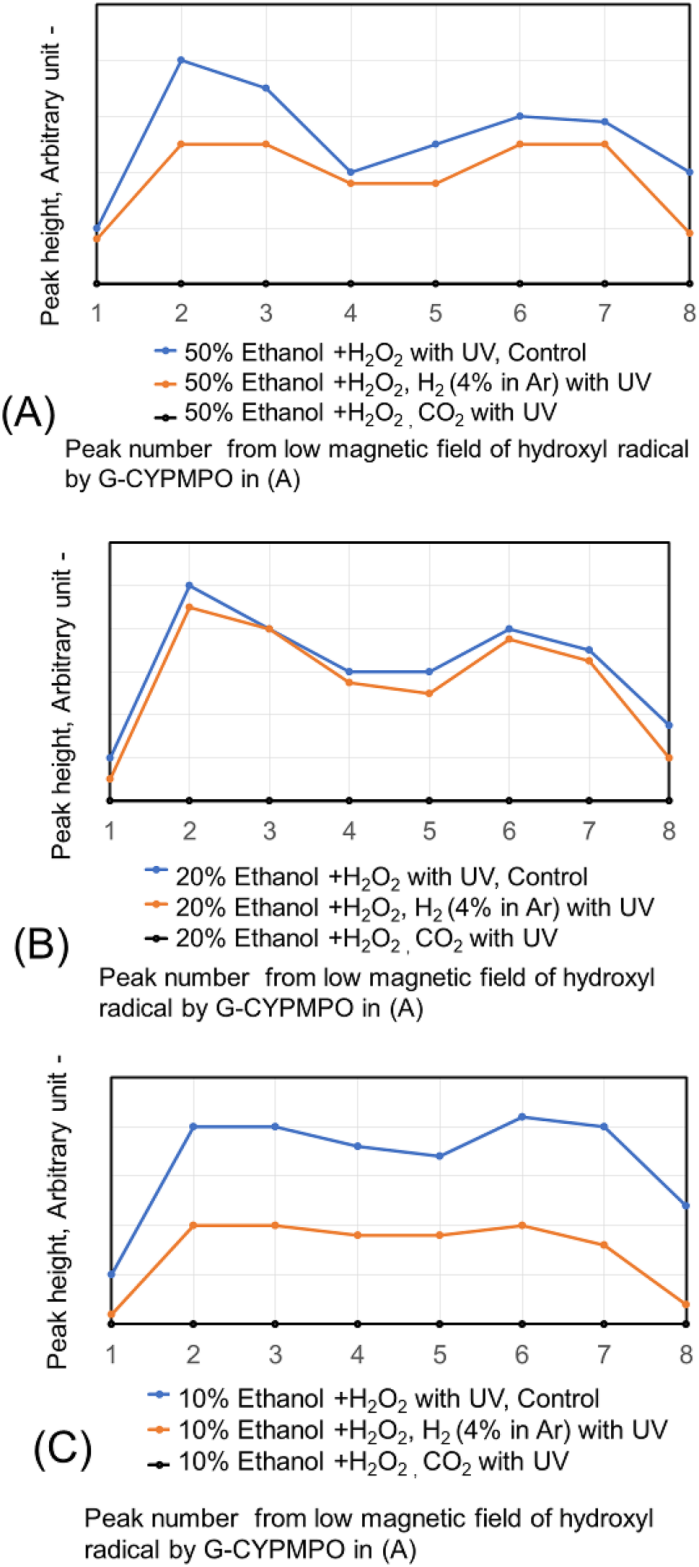
Peaks of hydroxyl radical with ESR by blowing CO_2_ and H_2_ (4% in Ar) gas nanobubble into 10, 20, and 50% ethanol with 0.1% H_2_O_2_ aqueous solution.

**Figure 5:**
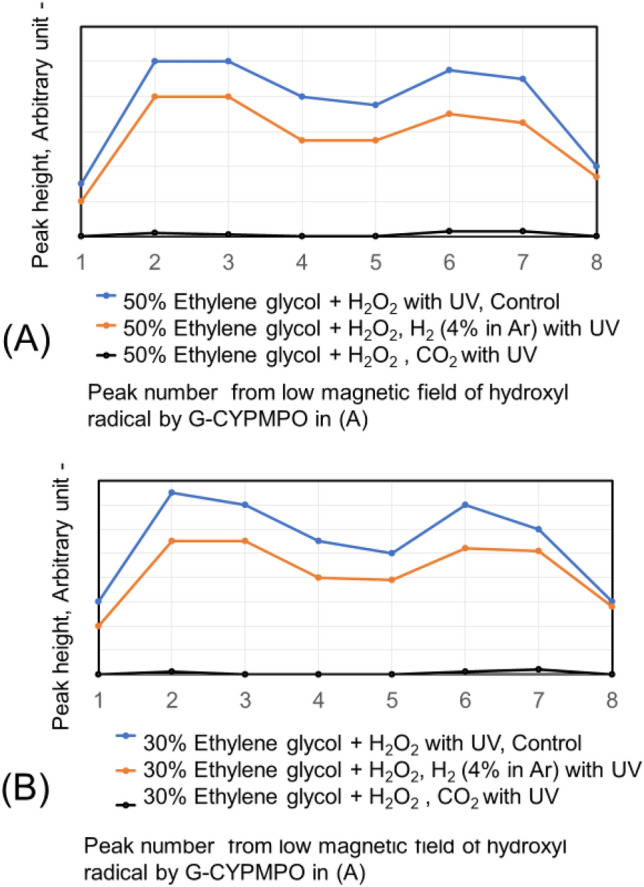
Peaks of hydroxyl radical with ESR by blowing CO_2_ and H_2_ (4% in Ar) gas nanobubble into 30 and 50% ethylene glycol with 0.1% H_2_O_2_ aqueous solution.

**Figure 6:**
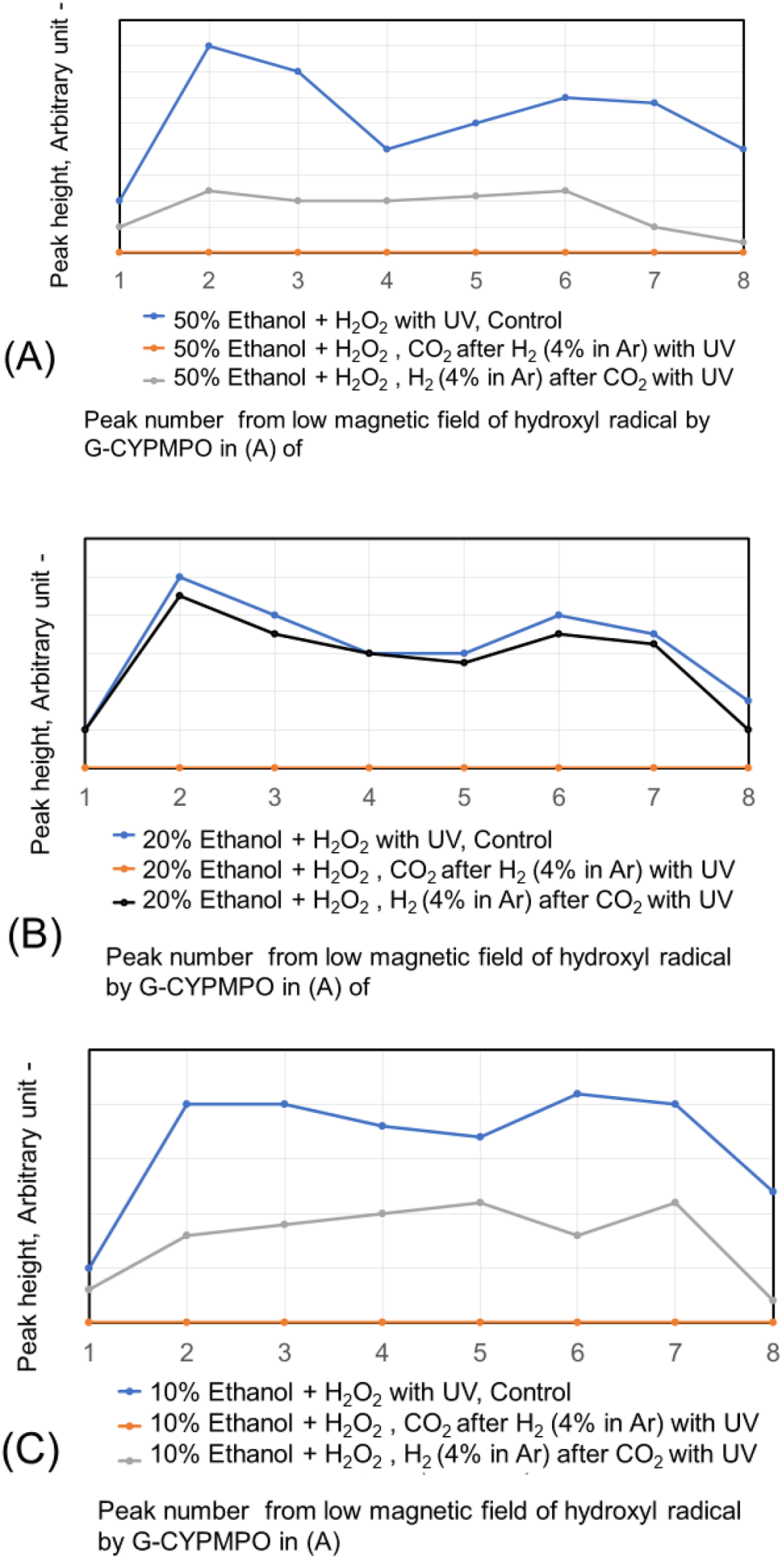
Peaks of hydroxyl radical with ESR by blowing CO_2_ after H_2_ (4% in Ar) gas nanobubble and H_2_ (4% in Ar) after CO_2_ gas nanobubble into 50, 20, and 10% ethanol with 0.1% H_2_O_2_ aqueous solution.

**Figure 7:**
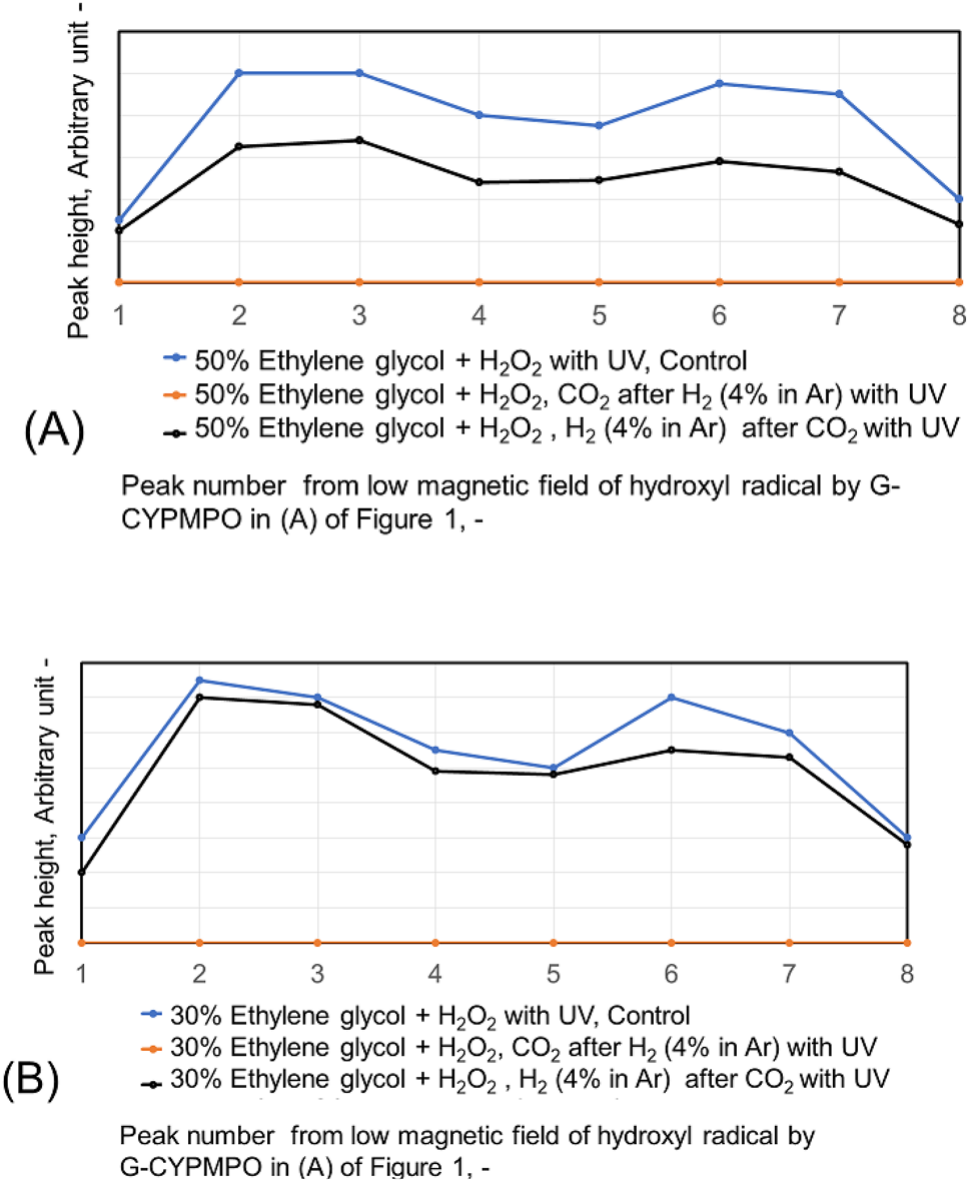
Peaks of hydroxyl radical with ESR by blowing CO_2_ after H_2_ (4% in Ar) gas nanobubble and H_2_ (4% in Ar) after CO_2_ gas nanobubble into 50 and 30% ethylene glycol with 0.1% H_2_O_2_ aqueous solution.

The CO_2_ and H_2_ (4% in Ar) are blown into 50%, 20%, and 10% of ethanol in water with 0.1% H_2_O_2_. The peaks of hydroxyl radical with ESR are shown in Fig. 4. The H_2_ (4% in Ar) gas bubbling reduced hydroxyl radical small. While the CO_2_ gas bubbling reduced hydroxyl radical almost completely in the used concentration of ethanol aqueous solution. The CO_2_ nanobubble in ethanol aqueous solution can eliminate the hydroxyl radical. The CO_2_ and H_2_ (4% in Ar) have been blown into 50% and 30% of ethylene glycol in water with 0.1% H_2_O_2_ as a dihydric alcohol aqueous solution and the peaks are shown in Fig. 5. The H_2_ (4% in Ar) gas bubbling can reduce the hydroxyl radical a little bit small, whereas the CO_2_ gas bubbling eliminates hydroxyl radical almost completely like the result of ethanol aqueous solution. Next, the hydroxyl radical degradation is investigated with the blowing order of CO_2_ and H_2_ (4% in Ar) gas. Peaks of hydroxyl radical with ESR by blowing CO_2_ after H_2_ (4% in Ar) gas and H_2_ (4% in Ar) after CO_2_ gas nanobubble into 50, 20, and 10% ethanol with 0.1% H_2_O_2_ aqueous solution are shown in Fig. 6. Only H_2_ (4% in Ar) blowing cannot decrease the hydroxyl radical peak as shown in Fig. 4, however, the CO_2_ blowing after H_2_ (4% in Ar) can disappear the hydroxyl radical peaks as shown in Fig. 6. On the other hand, only CO_2_ blowing can decrease the hydroxyl radical peaks as shown in Fig. 4, however, the H_2_ (4% in Ar) blowing increased the hydroxyl radical peaks again as shown in Fig. 6. For the 50 and 30% ethylene glycol aqueous solution the same phenomena have appeared. The peaks of hydroxyl radical with ESR by blowing CO_2_ after H_2_ (4% in Ar) gas nanobubble and H_2_ (4% in Ar) after CO_2_ gas nanobubble into 50 and 30% ethylene glycol with 0.1% H_2_O_2_ aqueous solution is shown in Fig. 7. The H_2_ (4% in Ar) blowing after CO_2_ increased the hydroxyl radical peaks again.

**Figure 8:**
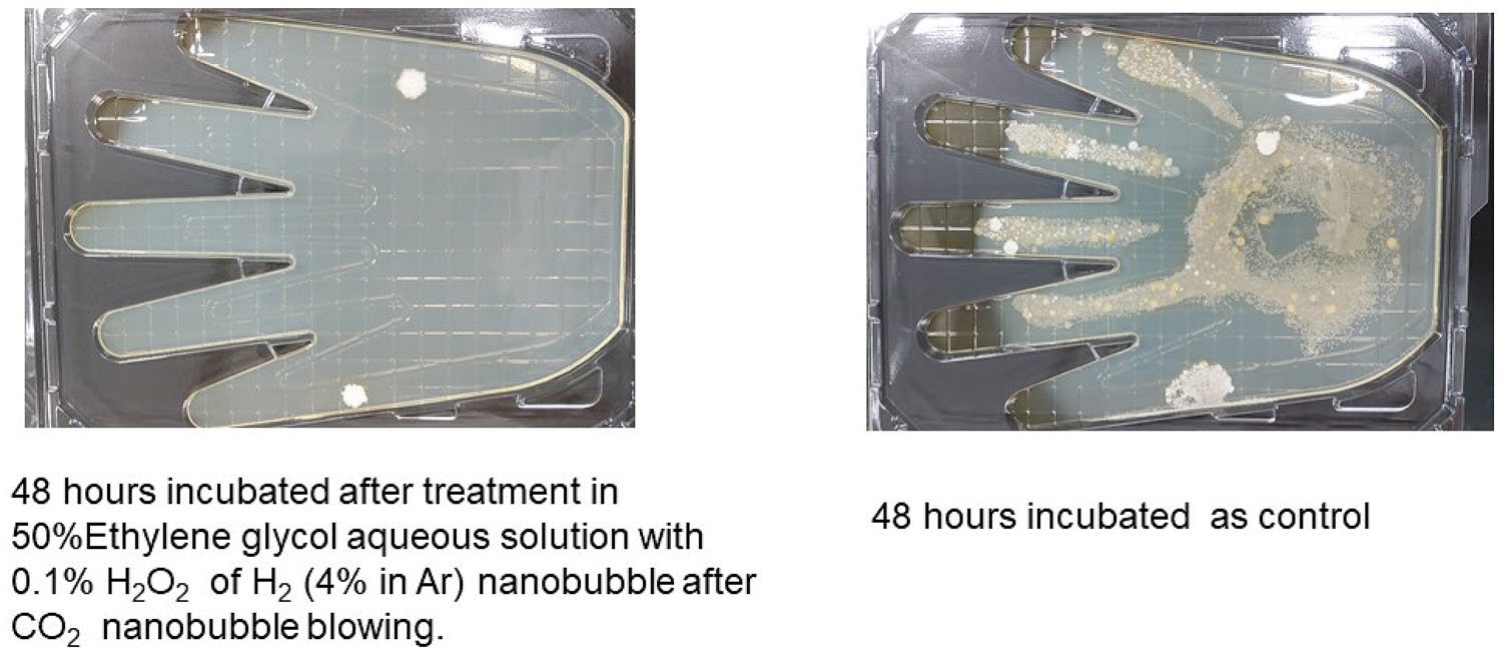
Photos of the plate are compared with the control and the solution containing nanobubbles treatment after 48 h of incubation.

Photos of the plate are compared with the control and the solution containing nanobubbles treatment after 48 h incubation is shown in Fig. 8. The 50% ethylene glycol with 0.1% H_2_O_2_ aqueous solution H_2_ (4% in Ar) nanobubble injection after CO_2_ gas nanobubble blowing showed no living bacteria after 48 h incubation. The hydroxyl radical in the solution as shown in Fig. 7 had an effect to prevent bacteria from propagating.

#### Superoxide anion radical degradation in alcohol aqueous solution

**Figure 9:**
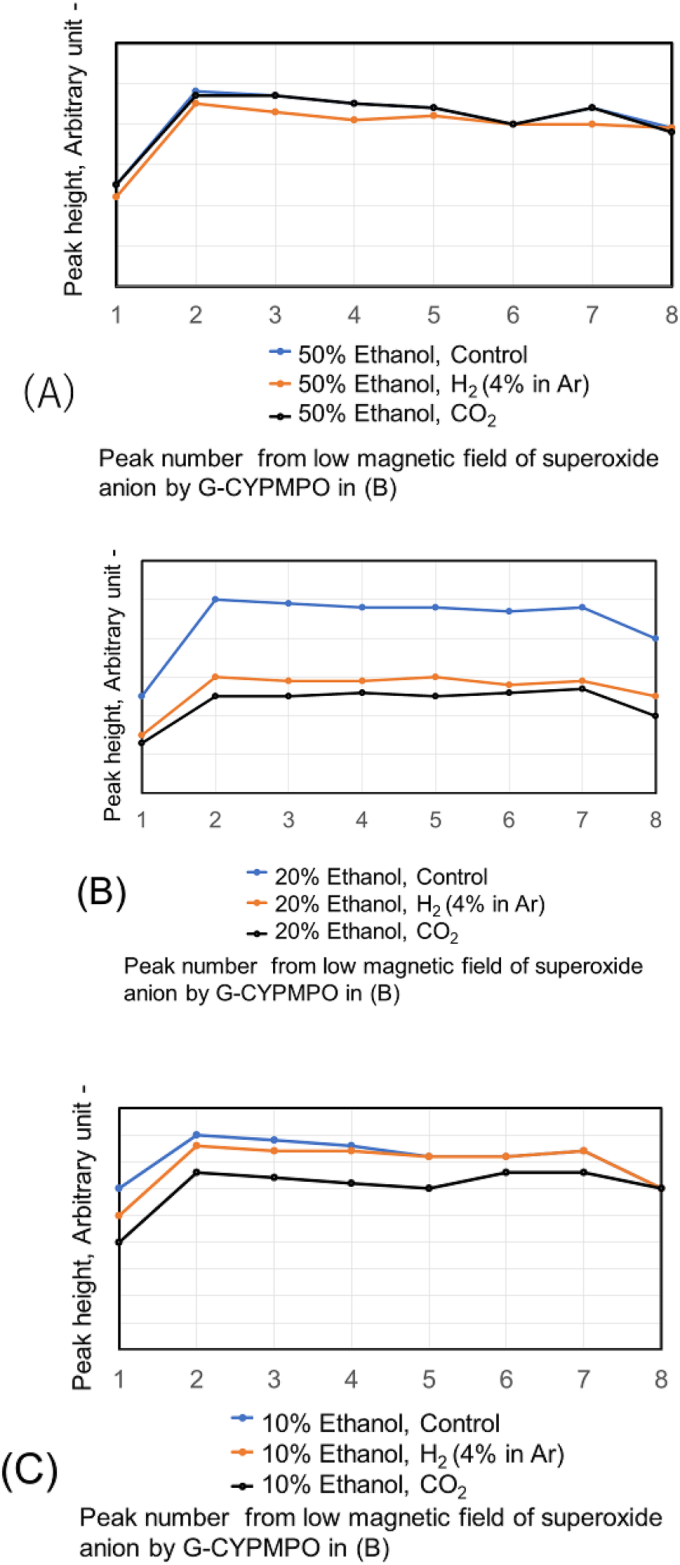
Peaks of superoxide anion radical with ESR in 10, 20, and 50% ethanol aqueous solution containing CO_2_ and H_2_ (4% in Ar) nanobubble.

**Figure 10:**
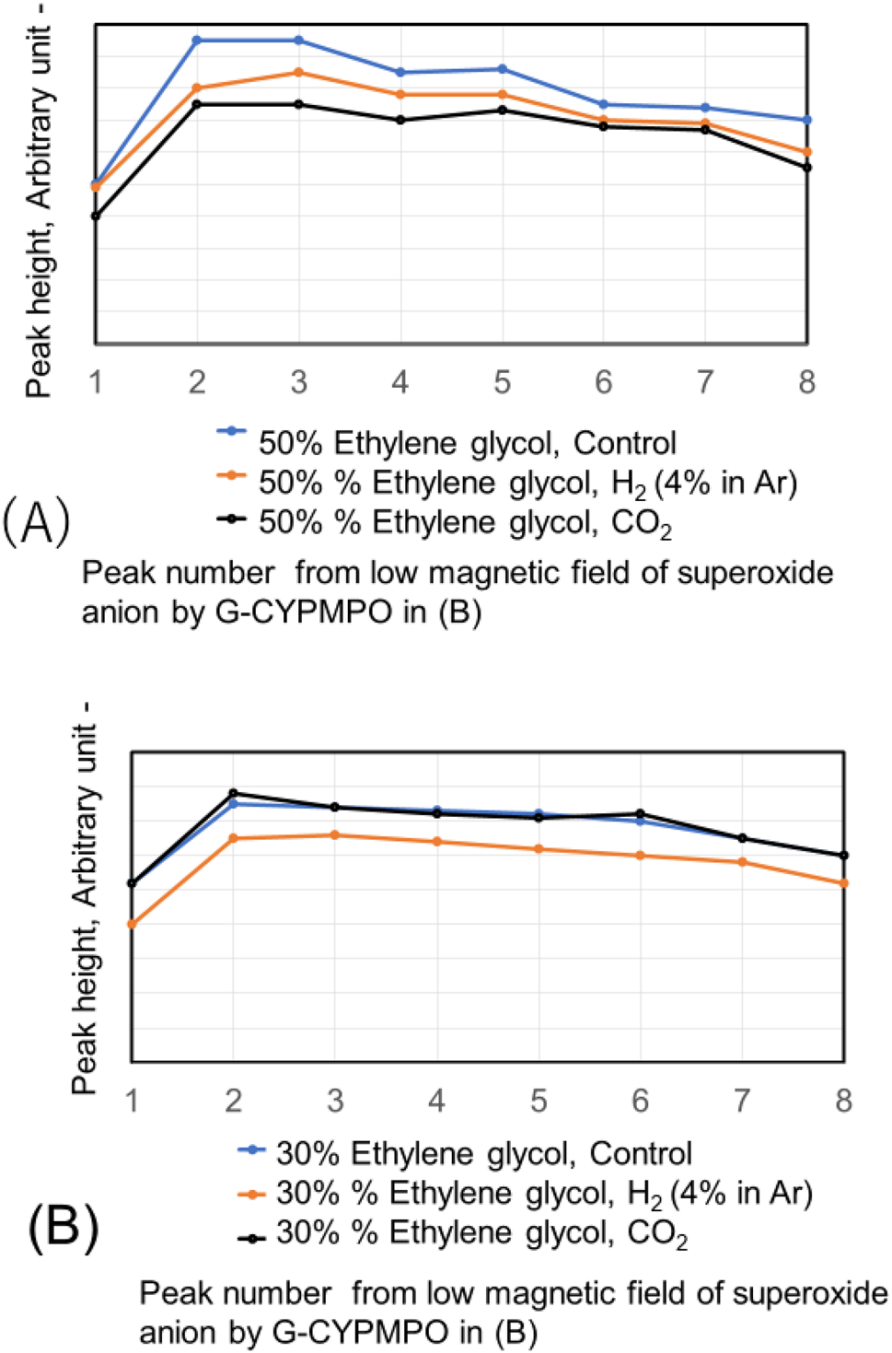
Peaks of superoxide anion radical with ESR in 30 and 50% ethylene glycol aqueous solution containing CO_2_ and H_2_ (4% in Ar) nanobubble.

**Figure 11:**
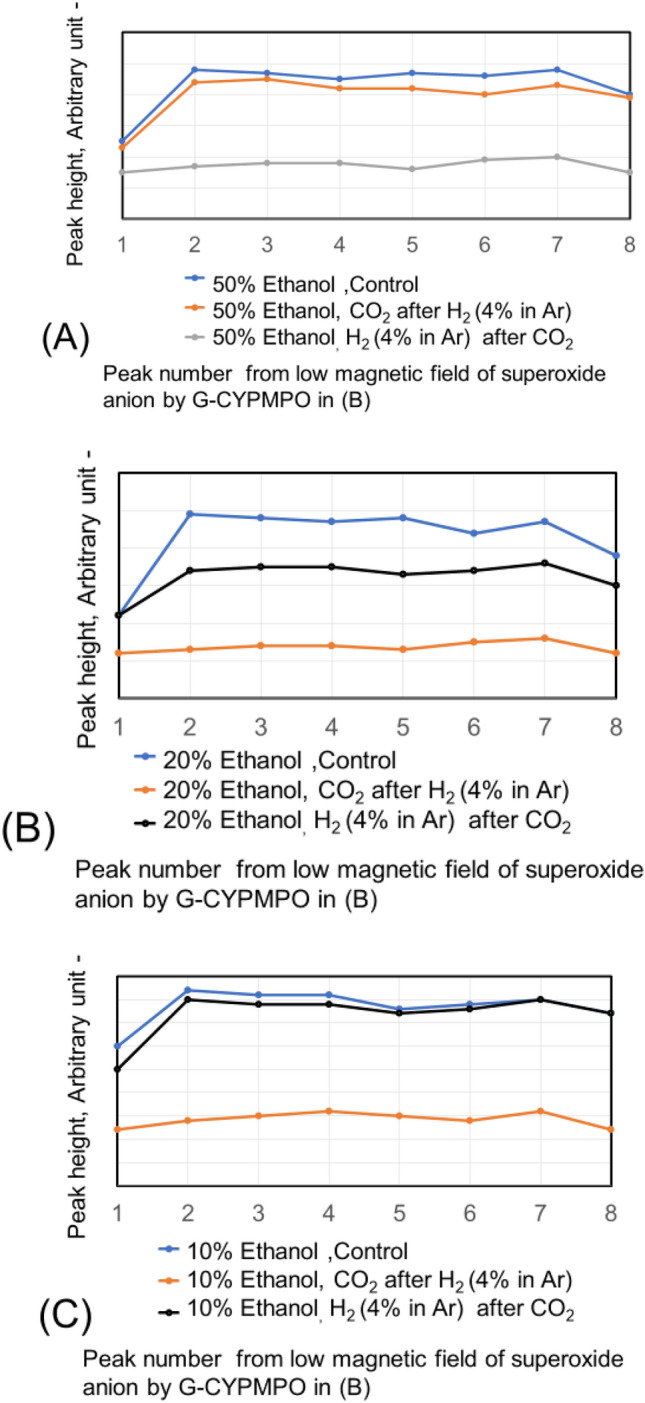
Peaks of superoxide anion radical with ESR by blowing CO_2_ after H_2_ (4% in Ar) gas nanobubble and H_2_ (4% in Ar) after CO_2_ gas nanobubble into 50, 20, and 10% ethanol aqueous solution.

**Figure 12:**
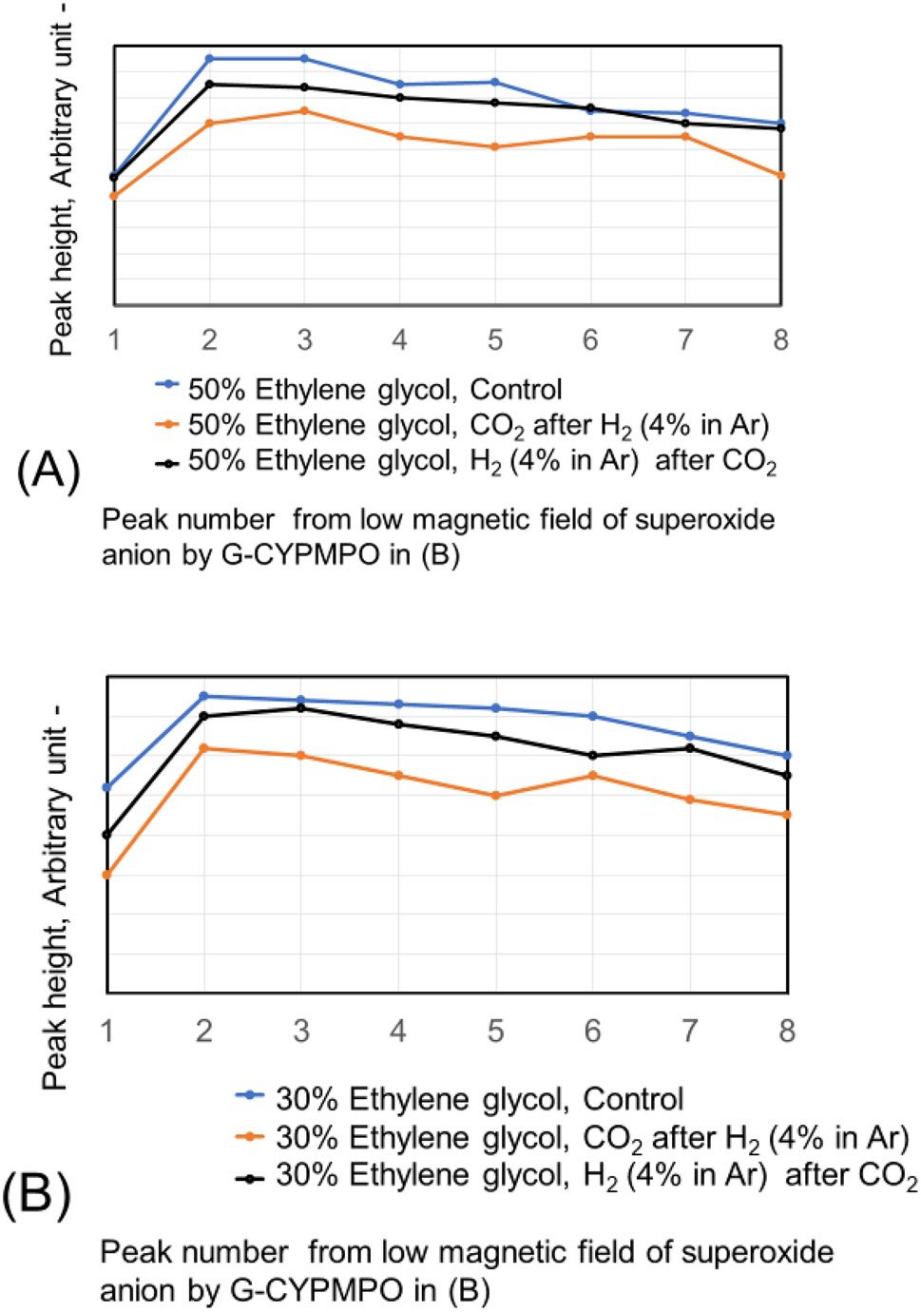
Peaks of superoxide anion radical with ESR by blowing CO_2_ after H_2_ (4% in Ar) gas nanobubble and H_2_ (4% in Ar) after CO_2_ gas nanobubble into 50 and 30% ethylene glycol aqueous solution.

The hypoxanthine (HX)/xanthine oxidase (XOD) solution is added and mixed well with 50%, 20%, and 10% of ethanol in water containing CO_2_ and H_2_ (4% in Ar) nanobubbles. Then the peaks of superoxide anion radical with ESR using spin trapping reagent of G-CYPMPO are shown in Fig. 9. The H_2_ (4% in Ar) gas and CO_2_ nanobubble included 10% and 20% ethanol aqueous solution reduced superoxide anion radical slightly. However, radical degradation cannot be seen in the 50% ethanol aqueous solution. The peaks of superoxide anion radical with ESR in 30 and 50% ethylene glycol aqueous solution containing CO_2_ and H_2_ (4% in Ar) nanobubble are shown in Fig. 10. For the 50 and 30% ethylene glycol aqueous solution, radical degradation is very small. The 30% ethylene glycol aqueous solution containing H_2_ (4% in Ar) nanobubble shows much smaller peaks, however, the CO_2_ peaks do not decrease. Next, the superoxide anion radical degradation is investigated with the blowing order of CO_2_ and H_2_ (4% in Ar) gas nanobubble in ethanol aqueous solution. The peaks of superoxide anion radical with ESR by blowing CO_2_ after H_2_ (4% in Ar) gas nanobubble and H_2_ (4% in Ar) after CO_2_ gas nanobubble into 50, 20, and 10% ethanol aqueous solution are shown in Fig. 11. Comparing the control, in the combination liquid of CO_2_ after H_2_ (4% in Ar) for 20 and 10% ethanol aqueous solution, the superoxide anion decreased comparing the peaks by only CO_2_ and H_2_ (4% in Ar) in Fig. 9. The ethylene glycol aqueous solution is investigated with the blowing order. The peaks of superoxide anion radical with ESR by blowing CO_2_ after H_2_ (4% in Ar) gas nanobubble and H_2_ (4% in Ar) after CO_2_ gas nanobubble into 50 and 30% ethylene glycol aqueous solution are shown in Fig. 12. The blowing of CO_2_ gas after H_2_ (4% in Ar) gas decreased larger than the H_2_ (4% in Ar) gas after CO_2_ gas for 50 and 30% ethylene glycol aqueous solution. This is a similar phenomenon to the 20 and 10% ethanol aqueous solution.

## Discussion

There are some reports for the diminishment of free radicals by hydrogen nanobubble as shown in the introduction session. The hydrogen nanobubble scavenged various ROS^18^ and reduced especially hydroxyl radical^15,17^. Oxygen nanobubble caused tissue damage^16^. In this experiment, the used solvents are pure water, ethanol aqueous solution as monohydric alcohol, and ethylene glycol aqueous solution as a dihydric alcohol mixture. The hydroxyl radical (∙HO) is produced by adding H_2_O_2_ in an aqueous solution and irradiation of UV as shown in the Eq. (1). The superoxide anion (∙O_2_^−^) was produced with an addition of hypoxanthine (HX) and xanthine oxidase (XO) in an aqueous solution. In the measurement of hydroxyl radical by ESR with UV, a large amount of CO_2_ and H_2_ nanobubbles are supplied into H_2_O_2_ added aqueous solution before measurement. While the superoxide anion was measured by ESR after the superoxide anion was mixed with O_2_ and H_2_ nanobubble containing aqueous solution. Some parts of CO_2_ and H_2_ nanobubbles are collapsed and the appeared gas seems to react to the radicals. There is a report that N_2_, O_2_ and CO_2_ nanobubbles produced by piston-type generator have gradually changed the size for 48 h^21^. After nanobubbles were produced, in a few days nanobubble size of N_2_, O_2,_ and H_2_ + Ar changes and becomes almost constant in a week and stable. However, the CO_2_ gas bubble size constantly changes larger and disappeared in several days. The nanobubble gas can supply the gas reacts to the radicals for several days. Several chemical formula^22^ of hydroxyl radical and superoxide anion radical reactions with hydrogen and oxygen are as follows,

1 $${\text{H}}_{2} {\text{O}}_{2} \,\,{\text{UV irradiation}} \to 2 \cdot {\text{HO}}$$

2 $$\cdot {\text{HO}} + {\text{CO}}_{2} \to \cdot {\text{CO}}_{3} {\text{H}}$$

3 $$\cdot {\text{CO}}_{3} {\text{H }} + {\text{H}}_{2} {\text{O}} \to {\text{H}}_{2} {\text{CO}}_{3} + {\text{ OH}}^{ - }$$

4 $${\text{H}}_{2} {\text{CO}}_{3} \Leftrightarrow {\text{HCO}}_{3}^{ - } + {\text{ H}}^{ + }$$

5 $${\text{H}}^{ + } + {\text{OH}}^{ - } \to {\text{H}}_{2} {\text{O}}$$

6 $$\cdot {\text{O}}_{2}^{ - } + {\text{ H}}_{2} \to 2 \cdot {\text{HO}}$$

7 $$\cdot {\text{HO}} + {\text{H}}_{2} {\text{O}}_{2} \to \cdot {\text{HO}}_{2} + {\text{ H}}_{2} {\text{O}}$$

8 $$\cdot {\text{HO}}_{2} \Leftrightarrow {\text{H}}^{ + } + \cdot {\text{O}}_{2}^{ - }$$

9 $$2{\text{H}}_{2} {\text{O}}_{2} \to 2{\text{H}}_{2} {\text{O}} + {\text{O}}_{2}$$

10 $${\text{H}}_{2} {\text{O}}_{2} + \cdot {\text{O}}_{2}^{ - } \to \cdot {\text{HO}} + {\text{OH}}^{ - } + {\text{O}}_{2}$$

11 $$2 \cdot {\text{O}}_{2}^{ - } + 2{\text{H}}^{ + } \to {\text{H}}_{2} {\text{O}}_{2} + {\text{O}}_{2}$$

12 $${\text{CH}}_{3} {\text{CH}}_{2} \left( {{\text{OH}}} \right) + \cdot {\text{O}}_{2}^{ - } + {\text{H}}_{2} \to \cdot {\text{HO}} + {\text{H}}_{2} {\text{O}} + {\text{CH}}_{3} {\text{CH}}_{2} \left( {{\text{O}}^{ - } } \right)$$

13 $${\text{HOCH}}_{2} {\text{CH}}_{2} {\text{OH}} + \cdot {\text{O}}_{2}^{ - } + {\text{H}}_{2} \to \cdot {\text{HO}} + {\text{H}}_{2} {\text{O}} + \left( {{\text{O}}^{ - } } \right){\text{CH}}_{2} {\text{CH}}_{2} {\text{OH}}$$

The decrease of the ESR spectrum of ∙HO in aqueous solution with CO_2_ nanobubble is shown in the reaction (2) → (3) → (4) → (5), therefore, ∙H O can be reduced. However, by blowing H_2_ (4% in Ar), O_2,_ and N_2_ nanobubble it is difficult to react directly to ∙HO in an aqueous solution. As shown in the ESR peaks of Fig. 2, only CO_2_ nanobubble can reduce the ∙HO in water. Also, the CO_2_ nanobubble can reduce ∙OH in the other alcohol aqueous solution as shown in Figs. 4 and 5. In Figs. 6 and 7, the CO_2_ blowing after H_2_ (4% in Ar) gas nanobubble showed a clear decrease in the peak of ∙HO similar to only CO_2_ blowing. On the other hand, the opposite mixing of H_2_ (4% in Ar) gas nanobubble after CO_2_ blowing did not decrease the peak of ∙HO and H_2_ (4% in Ar) nanobubble increased the peak of ∙HO. The produced ∙O_**2**_^**−**^ shown in the Eqs. (7) and (8) reacts with H_2_ and ∙HO is produced again by the reaction shown in Eq. (6). The reaction of ∙O_**2**_^**−**^ in aqueous solution with H_2_ (4% in Ar) nanobubble is shown in the reaction (6) and the ESR peaks of ∙O_2_^**−**^ with H_2_ nanobubble in water decreases slightly comparing with other O_2_, N_2,_ and CO_2_ nanobubble as shown in Fig. 3. Next, the ∙O_2_^**−**^ has mixed with water containing CO_2_ and H_2_ (4% in Ar) nanobubble. The produced ∙HO in (6) can be reduced by the CO_2_ nanobubble in the reaction of (2) → (3) → (4) → (5). When H_2_ (4% in Ar) nanobubble is blowing into ethanol and ethylene glycol with water, ∙HO is produced by the reaction (12) and (13). When the ∙O_2_^**−**^ has mixed with both CO_2_ and H_2_ (4% in Ar) nanobubble mixed solution, the peaks of.

∙O_2_^**−**^ has decreased clearly in the ethanol aqueous solution as shown in Fig. 10. While the peaks of ∙O_2_^**−**^ have decreased slightly in the ethylene glycol aqueous solution as shown in Fig. 11. The larger H_2_ nanobubble amount by H_2_ (4% in Ar) blowing after CO_2_ gas nanobubble might contribute to the reaction (6) → (7) → (8) and increase ∙O_2_^**−**^**.** The degradation of hydroxyl radical and superoxide anion as a free radical of active oxygen has been investigated. The CO_2_ nanobubble inclusion after H_2_ nanobubble injection into water and ethanol aqueous solution including hydroxyl radical and superoxide radical can reduce the free radical in aqueous solution and this phenomenon might contribute as a healthy beverage. On the other hand, the hydrogen nanobubble inclusion after CO_2_ nanobubble injection can increase the free radical in ethylene glycol aqueous solution shown in Fig. 7. When the ethanol and ethylene glycol containing H_2_O_2_ produces ∙OH with UV. The CO_2_ nanobubble decreases ∙OH by the reaction (2) → (3) → (4). Next remaining H_2_O_2_ produces ∙O_2_^**−**^ by the reaction (7) and (8). When ethanol and ethlenglycol exist with ∙O_2_^−^, H_2_ gas from H_2_ nanobubble generating by blowing H_2_ produces ∙OH as shown in (12) and (13). These phenomena will be useful to kill the bacteria, etc. and the photos in Fig. 8 have been indicated.

## Methods

### Nanobubble production

Nanobubbles are generated by blowing the different gas (4% H_2_ in argon (Ar), CO_2_, O_2,_ and N_2_) from the gas cylinder into distilled water, ethanol, and ethylene glycol aqueous solution through a porous ceramic sparger of 500 nm mean pore diameter for 30 min^23^. Next, the aqueous solutions containing H_2_ (4% in Ar) gas are blown CO_2_ gas again and the solution containing CO_2_ nanobubbles is blown H_2_ (4% in Ar) gas again to investigate the effect of nanobubble gas mixture. The nanobubble size distributions are measured by the dynamic light scattering (DLS) method (Otsuka Electronics Co., Ltd.). The prepared nanobubble solution is supplied in the radical experiment within 24 h. In this experiment, a 4% hydrogen in argon is used to produce the nanobubble for the security as the 4 vol % hydrogen is the lower explosive limit. The carbon dioxide from the gas cylinder is more than 99.5 vol %. The existing nanobubble percentage in water is about 0.1 vol% after blowing 30 min. The nanobubble is blown in the water, 50%, 20%, and 10% ethanol as monohydric alcohol in water and 50%, 30% ethylene glycol as dihydric alcohol in water.

### Radical production

The hydroxyl radical is produced with 0.1 wt% H_2_O_2_ addition in aqueous solution and 10-s ultraviolet irradiation. The superoxide anion was produced with a hypoxanthine (HX) and xanthine oxidase (XO) system. A mixture of 3.6 μL of 10.97 units/ml XO, 20 μl of 20 μM HX, 156.4 μL of the nanobubble sample solution, and 20 μl spin trapping reagent solution are mixed.

As spin-trapping reagent, sc-5-(5,5-dimethyl-2-oxo-1,3,2-dioxapho-sphinan-2-yl)-5 methyl-1-pyrroline N-oxide (G-CYPMPO)^24^ has been used. CYPMPO can spin-trap superoxide and hydroxyl radicals and the half-life for the superoxide adduct of CYPMPO produced in UV-illuminated hydrogen peroxide solution and HX/XO solution are about 15 min and 50 min, respectively^25^. The kinetic evaluation of spin trapping rate constants of CYPMPO was reported^26^. The 0.1 wt% H_2_O_2_ was added to the aqueous solution before blowing gas bubbles. While the superoxide anion was added after blowing the gas bubbles.

### Measurement of radical existence

JEOL JES-TE25X ESR spectrometer is used to obtain ESR spectra of free radicals of hydroxyl radical and superoxide anion. To investigate the decomposition of hydroxyl radicals by nanobubbles, the various gas has been blown into 0.1 wt% H_2_O_2_ added aqueous solution, and the spectra of hydroxyl radicals by ESR is measured. While to decompose the superoxide anion, the superoxide anion is added into the various nanobubbles included aqueous solution and the spectra of superoxide anion have been measured by ESR.

### Disinfection tests

Hand Petancheck II Tryptone Soya Agar Medium [Eiken Chemical Co., Ltd.]^27^ is used to measure the total number of living bacteria on hand. The hand is put into an aqueous solution containing viable bacteria and touch the plate. The plate is incubated for 48 h as control. The other plate is soaked with ethylene glycol aqueous solution containing nanobubble and after removing the solution the plate is incubated for 48 h. After 48 h the photos of the plate are compared with the control and the solution containing nanobubbles treatment.
